# Prevalence of and risk factors for depressive and anxiety symptoms in a large sample of Chinese adolescents in the post-COVID-19 era

**DOI:** 10.1186/s13034-021-00429-8

**Published:** 2021-12-27

**Authors:** Xiaobin Zhang, Haidong Yang, Jing Zhang, Man Yang, Nian Yuan, Junjie Liu

**Affiliations:** 1 Institute of Mental Health, Suzhou Psychiatric Hospital, The Affiliated Guangji Hospital of Soochow University, Suzhou, 215137 People’s Republic of China; 2 Department of Psychiatry, The Fourth People’s Hospital of Lianyungang, The Affiliated KangDa College of Nanjing Medical University, Lianyungang, 222003 People’s Republic of China; 3 Science and Education Section, The Fourth People’s Hospital of Lianyungang, The Affiliated KangDa College of Nanjing Medical University, Lianyungang, 222003 People’s Republic of China

**Keywords:** Depression, Anxiety, Prevalence, Risk factors, Adolescents, Post-COVID-19 era

## Abstract

**Background:**

Depressive and anxiety symptoms are widespread among adolescents today, creating a large social problem. However, few previous studies have addressed depression and anxiety among adolescents in Chinese cohorts. The aim of this study was to evaluate the prevalence of and risk factors for depressive and anxiety symptoms among Chinese middle school adolescent students in the post-COVID-19 era.

**Methods:**

A total of 22,380 middle school students from Jiangsu Province were surveyed online, and their general demographic data were collected. The Patient Health Questionnaire-9 (PHQ-9) was used to assess depressive symptoms, and the seven-item Generalized Anxiety Disorder (GAD-7) scale was used to measure anxiety symptoms.

**Results:**

Of these participants (aged 12–17 years), 25.6% had depressive symptoms, 26.9% had anxiety symptoms, and 20.6% had a combination of depression and anxiety symptoms. The prevalence of depressive symptoms was higher in female adolescents (27.6%) than in male adolescents (23.7%; χ^2^ = 45.479, P = 0.000), and the proportion with anxiety symptoms was higher among female adolescents (28.6%) than among male adolescents (25.4%; χ^2^ = 29.390, P = 0.000). Furthermore, binary logistic regression analysis showed that gender, region, and parental relationship were significantly associated with depressive symptoms among adolescents, while age, gender, region, and parental relationship were significantly associated with anxiety symptoms.

**Conclusions:**

Our findings demonstrated that the prevalence of reported depressive and anxiety symptoms in Chinese adolescents are high. Female gender, urban region, and poor parental relationship may be risk factors for depressive and anxiety symptoms. Furthermore, policy makers, schools, and families need to pay more attention to the psychological health of adolescents, develop response plans and take early intervention measures to reduce the prevalence of adolescent depression and anxiety.

## Background

Depressive and anxiety disorders are currently considered complex and common illnesses that have serious effects on patients’ livelihood due to an uncertain etiology and heterogeneous influencing factors that differ throughout the world. In 2016, major depressive and anxiety disorders were the fifth and ninth leading causes of years lived with disability (YLDs), respectively [1]. The Global Burden of Disease (GBD) study predicted that unipolar depressive disorders would be the second leading cause of GBD by 2030 [2]. In 2019, a study found that the percentages of global disability-adjusted life-years (DALYs) attributable to depressive disorders and anxiety disorders were ranked the 13th and 24th leading causes of disability, respectively, from the teenage years through old age in 2019, whereas the percentage of DALYs among adolescents aged 10–24 rose to fourth and sixth leading causes [3]. The prevalence of both depressive and anxiety disorders have been on the rise in recent years because of rapid social and economic development, unhealthy lifestyles including smoking, frequent drinking, physical inactivity, sleep deprivation, low fruit consumption, and psychological stress responses [4]. In particular, depression and anxiety have been more prevalent among adults and adolescents during the worldwide COVID-19 pandemic [5, 6].

Depression and anxiety may cause serious unfavorable health outcomes for adolescents now and later in life. A study of adolescents with depression reported that 10% of adolescents, in particular, 10.7% of 12- to 14-year-olds and 9.4% of 15- to 17-year-olds, presented at least one suicidal ideation or suicide attempt [7, 8]. More importantly, suicide has gained more concern worldwide, as it is the second leading cause of death among young people aged 15–29 years, and suicide mortality rates occurring in low- and middle-income countries are higher than those in high-income countries [9]. In addition, both bipolar depression and unipolar depression may increase the risk of suicidal attempts, drug abuse, anxiety disorders and co-occurring medical illnesses in adolescents [10]. Increasing evidence has suggested that there is a close relationship between chronic physical illness (such as chronic fatigue syndrome, epilepsy, sensory impairment and migraine or tension headache) and anxiety disorders in children and adolescents [11]. Therefore, it is of great significance to document the prevalence of and factors related to depression and anxiety disorders in this special age group.

There is great discrepancy in the prevalence of depressive and anxiety disorders across different countries and regions. Ghandour et al. reported that the prevalence of current depression and anxiety problems in the US was 6.1% and 10.5%, respectively, among adolescents aged 12–17 years in 2016 [12]. Data from the American national comorbidity survey–adolescent supplement (age 13–18 years) showed that the lifetime prevalence of anxiety disorders was 31.9% and that of mood disorders was 14.3% [13]. Existing data from the GBD 2010 and the GBD 2013 indicated that the global prevalence data for depression and anxiety disorders in individuals aged 5–17 years was 6.2% and 3.2%, respectively [14]. A systematic review and meta-analysis found that the pooled prevalence estimates of depression and anxiety (mean age of 6–25 years) were 14.3% and 19.1% in Economic Co-operation and Development countries between January 2000 and January 2018 [15]. Furthermore, another meta-analysis calculated that 41.7% and 34.5% of children aged ≤ 18 years suffered from depression and anxiety disorder, respectively, during lockdown and quarantine measures in response to the COVID-19 pandemic [16]. Previous studies found that the prevalence of depression ranged from 4 to 41%, with a pooled prevalence of 19.85%, in Chinese children and adolescents before the COVID-19 outbreak [17]. Unexpectedly, the percentage of Chinese adolescents with depressive symptoms reached 43.7% and the percentage with anxiety symptoms increased to 37.4% during the COVID-19 epidemic period [18]. In short, the prevalence of depression and anxiety symptoms has been examined in children and adolescents, but the findings have been inconsistent due to different factors, such as time periods, stressful events, economic contexts and sociocultural factors. In particular, stressful responses were shown to have an impact on adolescents’ sleep difficulties and depressive and anxiety symptoms in China [19].

Accordingly, an investigation is essential to assess the relationships between the prevalence of depressive and anxiety symptoms and sociodemographic factors among adolescents in the post-COVID-19 era. The objective of the present study was to evaluate the proportion and associated factors of depressive and anxiety symptoms among Chinese adolescent school students in the post-COVID-19 era. To our knowledge, few studies to date have investigated the prevalence and related factors of depression and anxiety among adolescents in the post-COVID-19 era in China.

## Methods

### Procedures and subjects

The present study was a cross-sectional survey performed from January to February 2021 in Lianyungang, Jiangsu Province, China, which went six months without new local confirmed cases during this period [20]. We invited students from seven junior high schools to conduct an online survey using the Wenjuanxing management platform (https://www.wjx.cn/app/survey.aspx). Seven junior high schools were randomly selected based on their district, and students in grades 7–9 were invited to participate in the survey. The project was approved by the Ethics Committee of the Fourth People's Hospital of Lianyungang City, and the investigation was approved by the school, parents and students. Before taking the online survey, students were given a statement detailing the purpose of the survey, the process, how the data will be used, confidentiality and anonymity. Their participation was voluntary, and the results were blind to parents and teachers. If students did not want to complete filling out the questionnaire, they could stop at any time. A total of 23,025 questionnaires were collected from seven junior high schools. A total of 645 (2.8%) surveys were excluded due to incomplete answers for the necessary information, such as age, gender, and the questionnaire content. Consequently, the ultimate sample for the present study included 22,380 adolescents.

### Measures

A homemade sociodemographic data questionnaire designed by research staff collected information including age, gender, grade, school location (urban/rural area), and parental relationship (harmony/conflict/single-parent family), for parental relationship, the categories of harmony and conflict indicate a two-parent family, while the single-parent family category includes harmony or conflict in a single-parent family. The seven junior high schools were classified as urban or rural areas based on their location.

The Patient Health Questionnaire-9 (PHQ-9) was used to measure the severity of depressive symptoms and is a pragmatic, empirical and simple self-assessment tool [21]. It comprised nine items that were rated at one of four levels used to assess the frequency of depression during the past 2 weeks: not at all, several days, more than half the days, or nearly every day. Each item was scored using a four-point Likert scale: 0 for “not at all”, 1 for “several days”, 2 for “more than half the days”, and 3 for “nearly every day”. The total score could range from 0 to 27; a score of 0–4 was considered minimal, a score of 5–9 was considered mild, score of 10–14 was considered moderate, score of 15–19 was considered moderately severe, and score of 20–27 was considered severe in terms of the recommendation of Kroenke et al. [21]. The Chinese version of the PHQ-9 showed good reliability and validity in both the general population and adolescents [22]. In the present study, the Cronbach’s alpha coefficient value for the PHQ-9 was 0.928.

Anxiety symptoms in the adolescents were measured by the Generalized Anxiety Disorder Scale-7 (GAD-7), which is a self-report scale that has good reliability and validity [23]. Based on Spitzer et al. [24], the GAD-7 has seven items, and the total scores can range from 0 to 21 points. A score of 0–4 was considered minimal, a score of 5–9 was considered mild, a score of 10–14 was considered moderate, and a score of 15–21 was considered severe. The questionnaire reflected the frequency and impact of anxiety symptoms over the last two weeks. Each item was scored on a 0–3 point scale: “not at all” was scored 0, “several days” was scored 1, “more than half the days” was scored 2, and “nearly every day” was scored 3. The Chinese version of the GAD-7 has proven to have good validity in evaluating anxiety symptoms [25]. The Cronbach’s alpha coefficient of the GAD-7 was 0.934.

### Statistical analysis

The dataset analyses were conducted by IBM SPSS version 22.0. The Kolmogorov–Smirnov test was used to test the normality of the distribution of the sociodemographic data of the participants. Independent-samples *t* tests were used to compare the differences between continuous variables across groups, and categorical variables were evaluated with chi-square tests. The prevalence of depressive and anxiety symptoms was evaluated using the chi-square test. Univariate analysis validated possible risk factors. There were two dichotomous dependent variables (presence versus absence of depression and presence versus absence of anxiety symptoms), and logistic regression was used to determine the relationship between depression and anxiety symptoms and the other variables to identify predictive factors. All calculated P values were 2-sided, and statistical significance was set at a level of < 0.05.

## Results

### Sociodemographic characteristic of participants

The overall prevalence of reported depressive symptoms, anxiety symptoms, and comorbid depression and anxiety symptoms were 25.6%, 26.9%, and 20.6%, respectively. As shown in Table 1, univariate analyses demonstrated that the proportions of students in junior grade one, junior grade two, and junior grade three were 34.7% (n = 7776), 40.1% (8977), and 25.1% (5627), respectively. The rates of the parental relationship being in harmony, in conflict, and a single-parent family were 77.3% (n = 17,305), 17.5% (n = 3923), and 5.1% (n = 1152), respectively.

**Table 1:** Sociodemographic and clinical characteristics of adolescents

	n	%	Depressive symptoms			Anxiety symptoms		
			n	%	P	n	%	P
Gender								
Male	11,809	52.8	2798	23.7		2998	25.4	
Female	10,571	47.2	2921	27.6	0.000	3024	28.6	0.000
Region								
Urban	6676	29.8	1813	27.2		1901	28.5	
Rural	15,704	70.2	3906	24.9	0.000	4121	26.2	0.001
Grade								
Junior grade one	7776	34.7	1919	24.7		1992	25.6	
Junior grade two	8977	40.1	2345	26.1		2501	27.9	
Junior grade three	5627	25.1	1455	25.9	0.085	1529	27.2	0.004
Parental relationship								
Harmony	17,305	77.3	3822	22.1		3846	22.2	
Conflict	3923	17.5	1440	36.7		1647	42.0	
Single-parent family	1152	5.1	457	39.7	0.000	529	45.9	0.000
Total	22,380	–	5719	25.6	–	6022	26.9	–

### Prevalence of depressive and anxiety symptoms among adolescents

Our results revealed a significant difference in the prevalence of reported depressive and anxiety symptoms between male and female students (depressive symptoms: 23.7% versus 27.6%, χ^2^ = 45.479, P = 0.000; anxiety symptoms: 25.4% versus 28.6%, χ^2^ = 29.390, P = 0.000), as presented in Table 1. The prevalence of reported depressive symptoms among adolescents was significantly higher in urban regions than in rural regions (27.2% versus 24.9%, χ^2^ = 12.849, P = 0.000), and anxiety symptoms were also more prevalent in urban areas than in rural areas (28.5% versus 26.2%, χ^2^ = 11.881, P = 0.001). There were no significant differences in the prevalence of depressive symptoms across different grades (24.7% versus 26.1% versus 25.9% in junior grades one, two, and three, respectively, χ^2^ = 4.930, P = 0.085); interestingly, the rate of anxiety symptoms in junior grade one was lower than junior grade two and junior grade three (25.6% versus 27.9% versus 27.2%, χ^2^ = 10.924, P = 0.004). Compared with the adolescents in harmonious families, those in families with conflict and single-parent families reported a higher proportion of depressive and anxiety symptoms (depressive symptoms: 22.1% versus 36.7% versus 39.7%, χ^2^ = 486.552, P = 0.000; anxiety symptoms: 22.2% versus 42.0% versus 45.9%, χ^2^ = 858.006; P = 0.000).

The proportion of the sample with the various levels of depressive symptom severity from mild to moderate to moderately severe to severe was 14.6%, 6.0%, 3.2%, and 1.7%, respectively; meanwhile, the proportion of the sample with anxiety symptoms at the different levels of severity from mild to moderate to severe was 16.0%, 6.9%, and 4.1%, respectively. Moreover, 10.9% of the adolescents presented with moderate to severe depression, and 11.0% presented with moderate to severe anxiety symptoms, as shown in Table 2.

**Table 2:** Proportion of the adolescents with the various depression and anxiety symptom severity levels

	Depressive symptoms		Anxiety symptoms		Comorbid depression and anxiety symptoms	
	n	%	n	%	n	%
Minimal	16,661	74.4	16,358	73.1	17,763	79.4
Mild	3273	14.6	3572	16.0	_	_
Moderate	1345	6.0	1534	6.9	_	_
Moderately severe	714	3.2	_	_	_	_
Severe	387	1.7	916	4.1	_	_
Moderate to severe	2446	10.9	2450	11.0	4617	20.6

### Correlation analyses of the prevalence of depression and anxiety symptoms among adolescents

Univariate analysis showed no statistical significance in the prevalence of depressive and anxiety symptoms across different grades (P > 0.05), and independent sample t tests demonstrated no significant difference among different ages for depressive symptoms (P = 0.427) and a significant difference for anxiety symptoms (P = 0.005). Therefore, grade and age were not included in the logistic regression analysis for depressive symptoms, and grade was not included in the logistic regression analysis for anxiety symptoms. As shown in Table 3, regarding depressive symptoms among adolescents, binary logistic regression demonstrated that being a female student and in an urban region were positively related to depressive symptoms (B = 0.204, p = 0.000, OR = 1.226, 95% CI: 1.154–1.303; B = 0.112, p = 0.001, OR = 1.118, 95% CI: 1.047–1.194, respectively). Meanwhile, compared with harmonious families, families with a conflicting parental relationship and single-parent families were significantly positively correlated with depressive symptoms (B = 0.841, p = 0.000, OR = 2.318, 95% CI: 2.048–2.623; B = 0.713, p = 0.000, OR = 2.039, 95% CI: 1.893–2.197). In terms of anxiety symptoms, binary logistic regression revealed that age (B = 0.039, p = 0.014, OR = 1.040, 95% CI: 1.008–1.073), female gender (B = 0.160, p = 0.000, OR = 1.174, 95% CI: 1.105–1.247), urban region (B = 0.095, p = 0.005, OR = 1.100, 95% CI: 1.030–1.174), family with conflict (B = 1.088, p = 0.000, OR = 2.969, 95% CI: 2.630–3.353), and single-parent family (B = 0.927, p = 0.000, OR = 2.526, 95% CI: 2.348–2.717) were positively associated with anxiety symptoms.

**Table 3:** Risk factors associated with the prevalence of depression and anxiety symptoms

	Depressive symptoms			Anxiety symptoms		
	OR	95% CI	P	OR	95% CI	P
Age				1.040	1.008–1.073	0.014
Gender						
Male	1			1		
Female	1.226	1.154–1.303	0.000	1.174	1.105–1.247	0.000
Region						
Rural	1			1		
Urban	1.118	1.047–1.194	0.001	1.100	1.030–1.174	0.005
Parental relationship						
Harmony	1			1		
Conflict	2.318	2.048–2.623	0.000	2.969	2.630–3.353	0.000
Single-parent family	2.039	1.893–2.197	0.000	2.526	2.348–2.717	0.000

## Discussion

To our knowledge, the present study is the first cross-sectional clinical investigation in reference to the prevalence of and risk factors for depressive and anxiety symptoms among adolescents aged 11–17 years on the Eastern seaboard of China in the post-COVID-19 era. The main findings of the present study are as follows: (1) the prevalence of reported depressive and anxiety symptoms in adolescents were lower than those in other studies during the COVID-19 pandemic but remained at high levels. (2) Female students, urban region students, and students in families with poor parental relationships had a higher prevalence of reported depressive and anxiety symptoms. (3) Gender, region and parental relationship were significantly positively correlated with depression and anxiety symptoms among adolescents; additionally, age was positively associated with anxiety symptoms.

The present study found that depression symptoms, anxiety symptoms, and comorbid depression and anxiety symptoms affected 25.6%, 26.9%, and 20.6% of adolescents aged 11–17, respectively. The epidemic of depressive and anxiety disorders among adolescents is ongoing, but the results have been inconsistent. For example, Xie et al. reported that 22.6% and 18.9% of students had depressive and anxiety symptoms, respectively, because of the impact of the COVID-19 outbreak [26]. Zhou et al. reported that psychological health problems were prominent among Chinese adolescents during the COVID-19 epidemic; 43.7%, 37.4%, and 31.3% of middle school students aged 12–18 years suffered from depression symptoms, anxiety symptoms, and depression combined with anxiety symptoms, respectively [18]. Notably, before the COVID-19 outbreak, a meta-analysis suggested that the total pooled prevalence of depressive symptoms was estimated to be 22.2% among Chinese children and adolescents [27], and 6.06% of 6- to 17-year-old students were observed to be affected by anxiety symptoms in Northeast China [28]. Similarly, one study found that 21% suffered from depression symptoms, and 19% suffered from anxiety symptoms in Austria during COVID-19, and these percentages were higher than those in previous epidemiological studies [29]. Hertz and Barrios reported that adolescents in the US are showing an erosion of mental health and increased suicidality at an alarming rate due to the impact of COVID-19 [30]. Several possible reasons for these differences included the age range, sample size, assessment method and duration of depressive and anxiety symptoms, economic development levels and cultural differences, as surveys are conducted in different countries and regions. However, the data alone should cause concern for adolescent cohorts. Although many previous studies have suggested that the outbreak of the COVID-19 epidemic has affected children and adolescents’ mental health [31], few studies have shown the prevalence of depressive and anxiety symptoms and correlated factors among adolescents in China in the post-COVID-19 era.

This current study found that the prevalence of reported depressive symptoms and anxiety symptoms were higher in female students than in male students. These results were consistent with earlier studies [32, 33]. Moreover, a meta-analysis showed that the gender difference emerged at 12 years of age and peaked in adolescence; the gender difference was associated with an odds ratio (OR) of 1.95 for major depression, and Cohen’s *d* was 0.27 for depressive symptoms [34]. A survey of US adolescents aged 13–18 found that female adolescents experienced a higher (two- to threefold) risk of major depression than male adolescents, and anxiety disorder (fourfold increased risk in females) was significantly associated with major depression [35]. Furthermore, female adolescents had higher comorbidity with anxiety disorder than male adolescents, and the emergence of dysfunctional thoughts, high levels of perceived social support, social problem solving, and emotion regulation may be associated factors of depressive episodes in female and male adolescents [36]. Tan et al. reported that female students had poorer mental health status and more suicidal ideation than male students among Chinese children and adolescents [37]. Liu et al. also reported that female adolescents experienced more anxiety than male adolescents [38].

Additionally, our findings demonstrated that the percentage of depressive and anxiety symptoms among adolescents living in urban areas was significantly higher than that among adolescents living in rural areas. A similar result was reported in a previous study; the proportion of emotional problems in developed provinces was much higher than that in underdeveloped provinces [39]. A previous meta-analysis found that the pooled prevalence of psychiatric disorders, including mood and anxiety disorders, was higher in urban areas [40]. Moreover, we also found that parental factors played a vital role in students with depressive and anxiety symptoms. Parental factors, including granting less autonomy, expressing less warmth, showing aversiveness, showing overinvolvement, having more interparental conflict and engaging in more monitoring, were associated with depression and anxiety in adolescents [41]. Moreover, family relationship improvements were shown to contribute to reducing depressive symptom scores among Chinese junior high school adolescents [42].

The present study identified gender, region and parental relationship as risk factors for depressive and anxiety symptoms among adolescents in China. In addition, age was also a risk factor for anxiety symptoms. These results were consistent with earlier studies. In particular, many factors could influence brain development and cognitive maturation during puberty, and variations in brain physiology are closely linked with mood and anxiety disorders. For example, gonadal steroid surges quickly contribute to alterations within the limbic system, and the prefrontal cortex matures gradually, enabling social behavior [43]. Gonadal hormone secretion emerged and affected the structure and function of neural circuits in the prefrontal cortex and stria terminalis across adolescence and may play an important role in anxiety, especially in the gender differences observed during adolescence [44]. Previous evidence showed that the prevalence of depressive symptoms was higher among urban adolescents [45]. Mrug et al. found that increased sodium overnight excretion and reduced potassium excretion rates could predict more severe depressive symptoms occurring 1.5 years later in urban areas among African American adolescents [46]. Furthermore, a lack of family cohesion may lead to comorbid depressive and anxiety symptoms among adolescents in China [47].

Accordingly, our findings have clinical and policy implications. Depressive and anxiety symptoms among adolescents have become a major public health issue and may continue to increase in the future, which may bring a very large burden to families and society and affect the psychosomatic health of adolescents. First, it is necessary to provide policy makers with reliable data to assess the high-risk groups among adolescents and present a strategy to reduce the exposure to risk factors for depressive and anxiety symptoms. Furthermore, mounting evidence suggests that intervention strategies including psychotherapy and medication can be implemented as soon as possible for adolescents with moderate to severe symptoms of depression and anxiety. Moreover, adolescent mental health identification and promotion programs should be implemented on a larger scale, and this could be done not only by clinicians but also by schools, teachers and families in the future.

It is worth noting that the present study has several limitations. First, the present cross-sectional study cannot conclude direct causality between depressive and anxiety symptoms and risk factors among adolescents. Second, we did not gather data on other risk factors related to depressive and anxiety symptoms among adolescents, such as personality traits, academic performance, cultural factors, sleep duration, electronic device use time, bullying, only child status, and gender discrimination. Furthermore, as the pandemic continues to rage around the world, it may have more far-reaching consequences; the stress of a renewed outbreak may still be an important stressful event, not just for adults, but also for teenagers. Third, the results may have introduced bias, such as over- or underestimations of depressive and anxiety symptoms, due to the nature of the online survey, and importantly, self-report assessment cannot demonstrate the true prevalence of depression and anxiety symptoms. Fourth, it is difficult for young people to evaluate the relationship between their parents, so it is necessary to better specify the type of relationship between parents when designing questionnaires for use in the future. Fifth, the adolescents who participated in the online questionnaire came from one province, and we did not obtain information in other regions, which warrants further assessment in our future studies.

## Conclusions

In summary, our findings showed that the prevalence of reported depressive and anxiety symptoms among Chinese adolescents remained high in the post-COVID-19 era, and gender, region, and parental relationship were risk factors for depression and anxiety symptoms. Simultaneously, our results suggest that policy makers, schools and families need to pay more attention to the mental health of Chinese adolescents, and it is necessary to reinforce the early identification of depression and anxiety disorders among adolescents and design prevention programs as an important public health goal.

## Data Availability

The datasets used and analyzed during the current study are available from the corresponding author on reasonable request.

## Abbreviations

GBD: Global burden of disease
DALYs: Disability-adjusted life-years
COVID-19: Coronavirus disease 2019
PHQ-9: The Patient Health Questionnaire-9
GAD-7: The Generalized Anxiety Disorder Scale-7
OR: Odd ratio
CI: Confidence interval
